# Exploring surface electromyography (EMG) as a feedback variable for the human-in-the-loop optimization of lower limb wearable robotics

**DOI:** 10.3389/fnbot.2022.948093

**Published:** 2022-10-06

**Authors:** Martin Grimmer, Julian Zeiss, Florian Weigand, Guoping Zhao

**Affiliations:** ^1^Lauflabor Locomotion Laboratory, Department of Human Sciences, Institute of Sports Science, Technical University of Darmstadt, Darmstadt, Germany; ^2^Department of Electrical Engineering and Information Technology, Institute of Automatic Control and Mechatronics, Technical University of Darmstadt, Darmstadt, Germany

**Keywords:** EMG, human-in-the-loop, optimization, control, exoskeleton, wearable robotics, feedback, electromyography

## Abstract

Human-in-the-loop (HITL) optimization with metabolic cost feedback has been proposed to reduce walking effort with wearable robotics. This study investigates if lower limb surface electromyography (EMG) could be an alternative feedback variable to overcome time-intensive metabolic cost based exploration. For application, it should be possible to distinguish conditions with different walking efforts based on the EMG. To obtain such EMG data, a laboratory experiment was designed to elicit changes in the effort by loading and unloading pairs of weights (in total 2, 4, and 8 kg) in three randomized weight sessions for 13 subjects during treadmill walking. EMG of seven lower limb muscles was recorded for both limbs. Mean absolute values of each stride prior to and following weight loading and unloading were used to determine the detection rate (100% if every loading and unloading is detected accordingly) for changing between loaded and unloaded conditions. We assessed the use of multiple consecutive strides and the combination of muscles to improve the detection rate and estimated the related acquisition times of diminishing returns. To conclude on possible limitations of EMG for HITL optimization, EMG drift was evaluated during the Warmup and the experiment. Detection rates highly increased for the combination of multiple consecutive strides and the combination of multiple muscles. EMG drift was largest during Warmup and at the beginning of each weight session. The results suggest using EMG feedback of multiple involved muscles and from at least 10 consecutive strides (5.5 s) to benefit from the increases in detection rate in HITL optimization. In combination with up to 20 excluded acclimatization strides, after changing the assistance condition, we advise exploring about 16.5 s of walking to obtain reliable EMG-based feedback. To minimize the negative impact of EMG drift on the detection rate, at least 6 min of Warmup should be performed and breaks during the optimization should be avoided. Future studies should investigate additional feedback variables based on EMG, methods to reduce their variability and drift, and should apply the outcomes in HITL optimization with lower limb wearable robots.

## 1. Introduction

The human lower limb is required to execute a variety of movement tasks including locomotion as our most essential means of transport. Physical and functional limitations due to age or disease can limit lower limb-related mobility (Grimmer et al., 2019b). Furthermore, repetitive movement tasks or those movement tasks with heavy loads, such as lifting or carrying objects, can physically strain the human lower limbs and lead to fatigue and related injuries (Cavuoto and Megahed, 2017).

To overcome such mobility limitations and to reduce the impact of muscle fatigue, powered wearable assistive robotics such as lower limb prostheses (Windrich et al., 2016) and lower limb exoskeletons (del Carmen Sanchez-Villamañan et al., 2019; Pinto-Fernandez et al., 2020) were introduced. A variety of control concepts have been explored for these wearable robotics to assist users as much as possible in diverse terrains (Tucker et al., 2015; Pinto-Fernandez et al., 2020). Assistance timings and amplitudes were inspired by human biomechanics, such as joint moments and joint power trajectories (Grimmer et al., 2019a). While worldwide efforts have led to significant improvements in user assistance, many assistive device concepts have only been able to demonstrate minor benefits for the task of interest (Sawicki et al., 2020). One major reason for this is that conventional assistance control concepts are based on a certain generalized logic, and even though when hand-tuned by an expert, the majority of cases will have sub-optimal assistance timings and amplitudes.

To achieve optimal assistance, the assistance target variable, a metric that reflects performance, and its intended direction of change must first be defined. It also must be possible to measure the target variable while using the wearable device. Target variables could be related to user effort, gait symmetry (e.g., assisting with unilateral gait disorders), or biological peak loads (e.g., avoiding a maximum joint angle, moment, or power). Combinations of multiple and weighted target variables are possible. When the target variable is known, it is incorporated into a method known as human-in-the-loop (HITL) optimization (Koller et al., 2016; Zhang et al., 2017). With this method, the assistance timing and amplitude are stepwise-optimized online with the help of a feedback loop and with the aim of determining optimal individual assistance settings.

While this concept is quite promising, it currently suffers from some limitations. An approach must be developed to avoid having every movement task and variation require its own optimization (Han et al., 2021). For example, locomotion may require optimization for the bandwidth of gaits, terrain slopes, and speeds. Another major limitation is related to the feasibility of measuring the target variable. While it is difficult to measure or estimate target variables such as the biological moment or power, other variables are not practical for use in daily life. For example, using metabolic cost as a variable would require a mask to measure the human gas exchange. Furthermore, measuring metabolic cost has a limiting time constraint as it required 2 min of walking to obtain a reliable feedback variable for one assistance pattern (Zhang et al., 2017; Ding et al., 2018). Therefore, only 30 assistance patterns per hour could be explored and this could limit higher dimensional parameterizations (Ding et al., 2018).

Next to the metabolic cost, another option to estimate the user effort is heart rate. While this is easy to measure in daily life, its primary disadvantage is that not only the heart rate but also the stroke volume (which can not be measured easily), define the blood flow and, thus, the oxygen and energy supply to the human body (Higginbotham et al., 1986). As the behavior of the stroke volume is not linear with respect to oxygen consumption, the feasibility of measuring small changes in human effort could be limited.

A third option to determine human effort is to use the measured muscle activity (electromyography, EMG) of the involved muscles. EMG has been shown to increase with increased effort in both dynamic conditions, due to walking speed increase, and static conditions, due to muscle force increase against a fixed element (Onishi et al., 2000; Hof et al., 2002; Kuriki et al., 2012). Non-invasive surface electrodes can be used to acquire the required signal from lower limb muscles. A variety of robotic applications have used EMG as a control source (Singh et al., 2012; Rodríguez-Tapia et al., 2020). In a single case study, surface EMG signals showed promising results when used as a feedback variable in HITL optimization (Zhang et al., 2017). In Han et al. (2021), surface EMG was also proposed as a way to increase the number of testable parameter sets per hour. A trial duration of 1 min was used for EMG-based optimization, which doubles the number of testable variable sets per hour compared to using metabolic cost as a feedback variable. Without providing any explanation, the authors stated that even 40 s could be enough (Han et al., 2021) per iteration.

In this article, we aim to further investigate the fundamentals of using EMG within HITL optimization for lower limb wearable robotics such as prostheses and exoskeletons. Instead of using a wearable robot with all of its difficulties for the required perturbation, a laboratory experiment was designed to elicit changes in the human effort by picking up or releasing weight by the subjects during walking at a fixed speed on a treadmill. After picking up a weight, we expect, as previously seen by Browning et al. (2007), an increase in EMG amplitudes for the involved muscles to compensate for the additional walking effort. In contrast, after releasing the weight, reductions in EMG amplitudes should be seen while walking. If it is possible to distinguish between the loaded and unloaded walking conditions with a high detection rate (100% if every loading and unloading is detected accordingly), it should also be possible to use EMG for HITL optimization.

This study had the following objectives: (A) To be able to compare the loaded and unloaded EMG data, we first aimed to exclude the data from the transition phase between steady-state loaded and unloaded walking conditions. (B) To determine if multiple consecutive strides can improve the detection rate in between the conditions of loaded and unloaded walking compared to a single stride. (C) To determine if a combination of muscles can help distinguish the loaded and unloaded walking. (D) To investigate EMG drift over long periods of time for the same weight conditions as this could limit condition comparisons within HITL optimization.

(A) When changing the assistance pattern of a lower limb wearable robot, users must first react to the sudden perturbation due to a change in the assistance pattern and then restore a stable and steady gait. We believe that the transition phase should be excluded from the data comparison within HITL optimization, as in most cases, the user's muscle response will differ from a steady gait. This study is not designed to evaluate exactly how many strides are required for the transition. However, we aim to exclude the transition strides from the following analyses. We believe that a couple of strides should be sufficient to return to a steady gait with constant levels of EMG and EMG variability.

(B) For HITL optimization, it is of interest to be able to identify differences in the user response between assistance patterns, in our case based on the human effort determined by EMG. A change in human effort during gait is typically determined by the net metabolic cost (Sawicki et al., 2020) where maximum achieved reductions reach approximately 20% for autonomous exoskeletons (Lim et al., 2019) and 50% for tethered systems (Bryan et al., 2021). However, an increase in metabolic cost of 1.1–2% has been found from adding 1 kg of mass to the waist (Browning et al., 2007; Silder et al., 2013), though it is also difficult to distinguish such small changes in metabolic cost. In Browning et al. (2007), while a small increase in the net metabolic cost was found, no significant difference could be identified when adding 4 kg at the waist during walking, though significant differences were seen when 8 kg was added. We assume this to be attributed to metabolic cost signal variability. Also, EMG was found to have stride-to-stride variability (intra) and subject variability (inter), where either variability could be due to the biological variability of the central nervous system and the residual noise (Baratta et al., 1998).

Due to the expected EMG variability and based on the findings of the net metabolic cost of Browning et al. (2007), we decided to increase the subject weight by 2, 4, and 8 kg to investigate if it is possible to distinguish lower limb EMG of loaded and unloaded conditions. The detection rates were calculated. We hypothesize that it is possible to distinguish between loaded and unloaded conditions based on the mean average value of the EMG. Furthermore, due to EMG variability, higher detection rates should be achieved by comparing EMG data from multiple consecutive strides. Required data acquisition times to determine the detection rate point of diminishing returns for HITL optimization were estimated.

(C) When exploring assistance patterns with lower limb wearable robots the resulting effects on the lower limb muscles are unknown. While some muscles might be assisted, others could require increased effort to compensate for overshooting joint biomechanics or the resistance of the wearable device. Thus, it seems prudent to not only look at involved agonists but to also include antagonists for HITL optimization. Furthermore, as the function of the lower limb joints is closely coupled and dependent on each other, we expect a change in the function of one lower limb joint to induce additional changes in other lower limb joints and muscles and both limbs. For example, when assisting the hip with an exoskeleton the EMG of the soleus and gastrocnemius muscles changed and when assisting the ankle the EMG of the vastus and rectus femoris muscles changed (Franks et al., 2021). We hypothesize that higher detection rates for loading and unloading should be achieved by the combination of EMG data from multiple muscles.

(D) If changes in EMG amplitude due to signal drift are larger than the changes in amplitude due to the change in walking effort, this could limit the use of EMG within HITL optimization. Thus, knowing the drift will help to specify how to possibly use EMG for HITL optimization. Based on reported changes in EMG due to sweat, temperature, adaptations in walking biomechanics, and other reasons (Vøllestad, 1997; Day, 2002; Stewart et al., 2003; Abdoli-Eramaki et al., 2012; Meyer et al., 2019; Barsotti et al., 2020; Eken et al., 2020), we hypothesize that such drift exists with largest amplitudes during the Warmup and that reductions in amplitude occur over the course of the experiment.

## 2. Materials and methods

### 2.1. Subject information

**Figure 1:**
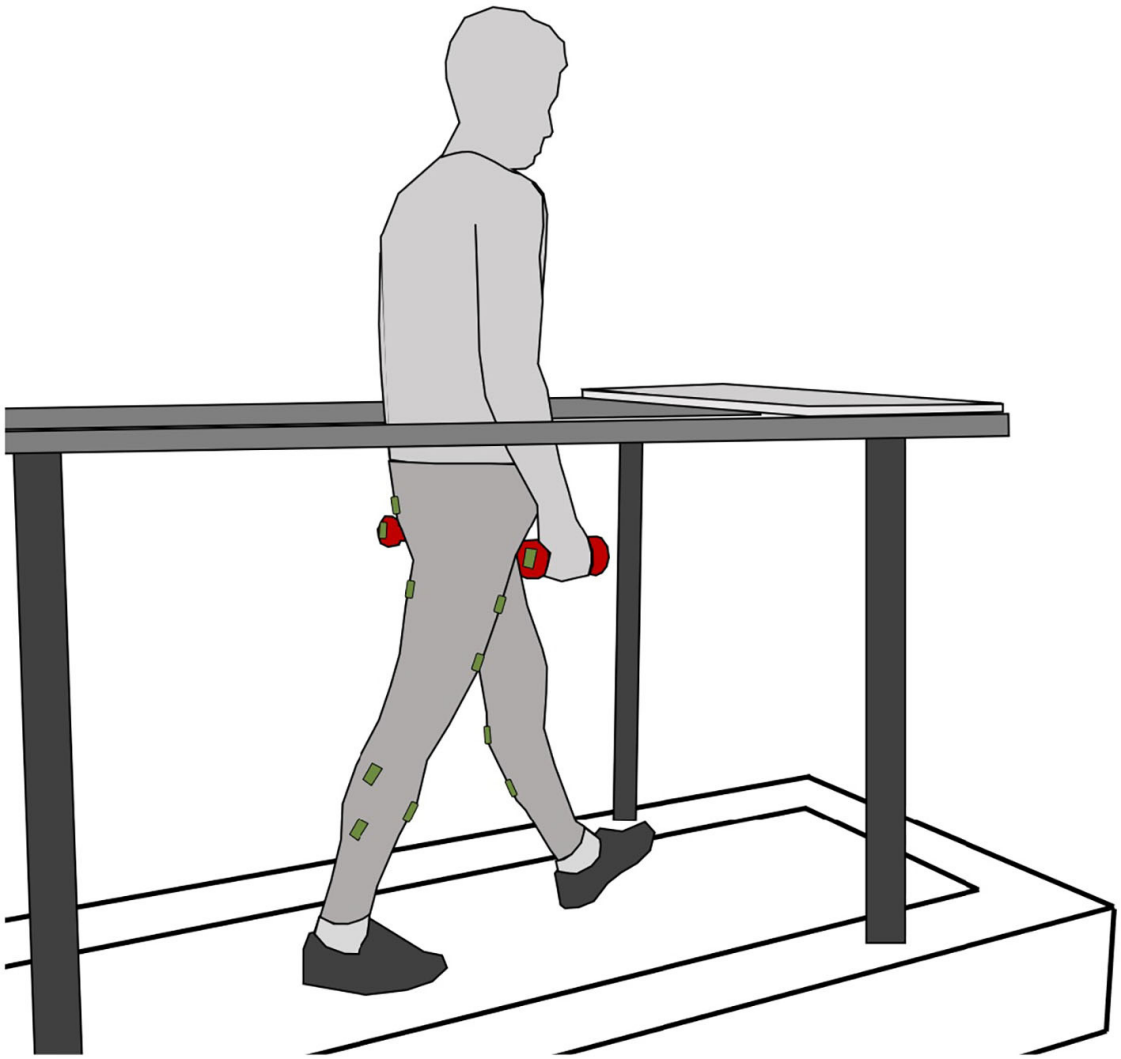
Subject on the treadmill while walking with one vinyl-coated dumbbell in each hand (red). The plate in front of the treadmill on the handrail is used to pick up or release the weights. Seven sensor units including EMG and gyroscopes were placed on the skin of each leg (green). One sensor unit was used only as a gyroscope and placed on each weight.

This study recorded and analyzed the EMG of the lower limbs of 13 subjects (27 ± 5 yrs, 1.82 ± 0.07 m, 81 ± 11 kg) during treadmill walking (Figure 1). Based on the verbal confirmation, all subjects were free of gait-related impairments. The study protocol was approved by the institutional review board of the Technical University of Darmstadt, Germany. All subjects provided written informed consent in accordance with the Declaration of Helsinki.

### 2.2. Experimental setup

**Figure 2:**
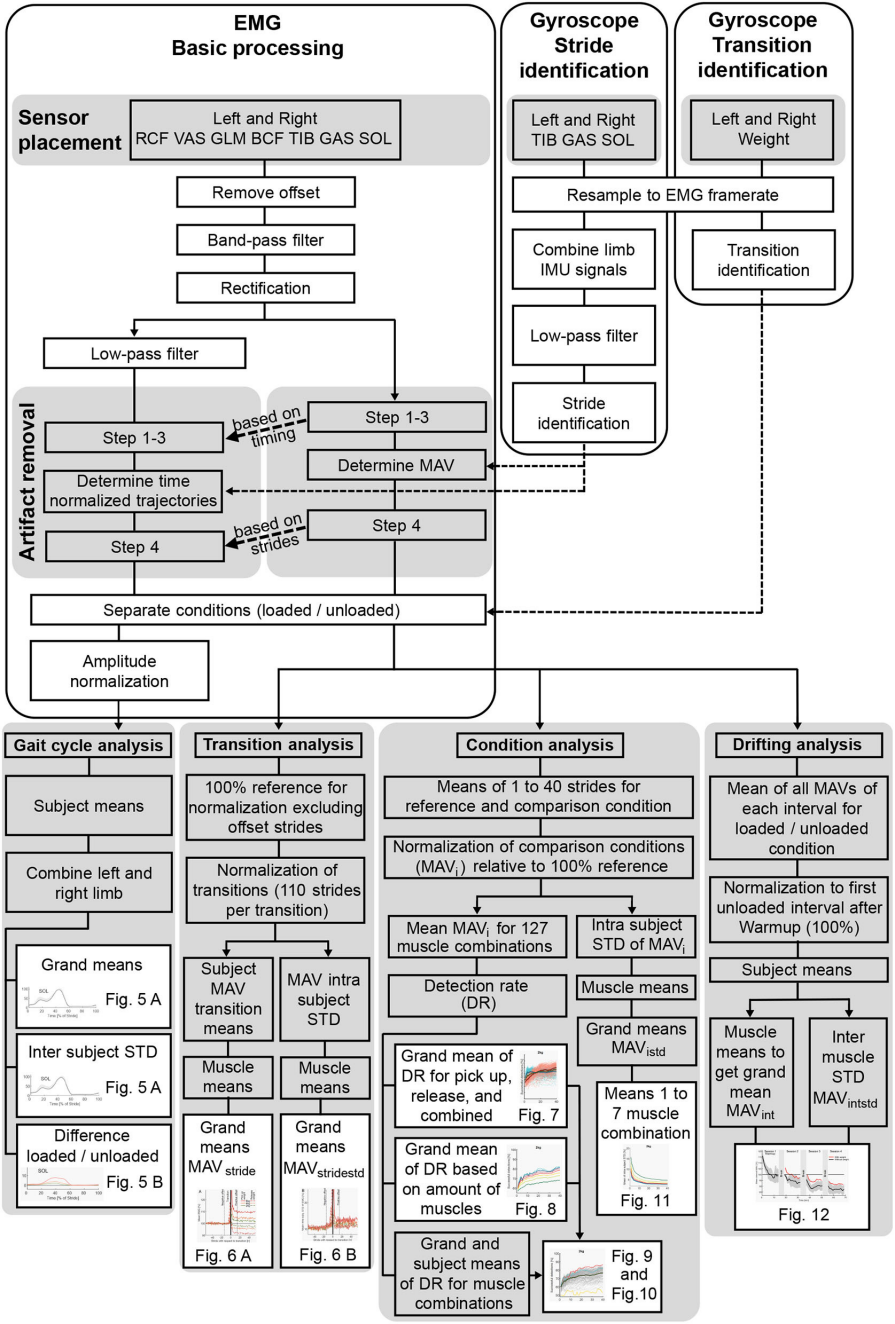
Overview of the data processing from the extracted signals to the use of the computed variables in the figures.

Lower limb EMG data was recorded at 1,926 Hz for seven muscles of each limb including the rectus femoris (RCF), vastus lateralis (VAS), glutaeus maximus (GLM), biceps femoris (BCF), tibialis anterior (TIB), soleus (SOL), and gastrocnemius lateralis (GAS). The wireless EMG sensors (Trigno Avanti, Delsys, Natick, MA, US) were placed based on the recommendations of SENIAM (seniam.org). In preparation for sensor fixation, hair was removed from the skin and the skin was cleaned with alcohol. To reduce the chance of loosening due to sensor movement and sweating, sensors were additionally affixed with adhesive non-woven fabric tape (Rudavlies). Each EMG sensor also included a 3D gyroscope (148 Hz). Gyroscopes of the TIB, GAS, and SOL were used to identify individual strides for each limb (Figure 2). One 3D gyroscope was also affixed to each of the vinyl-coated dumbbells to identify the timing of either picking up or releasing the weights. The study was carried out on a treadmill (ADAL-WR, HEF Tecmachine, Andrezieux Boutheon, France).

### 2.3. Experimental protocol

The study consisted of four main experimental sessions with each lasting 14 min. During each session, treadmill walking (with sportive shoes) was performed at a constant speed of 1.3 m/s, which is the preferred walking speed for adults (Grimmer et al., 2019b). The first session was always considered to be a Warmup session, in which data was only used in the drift analysis.

**Figure 3:**
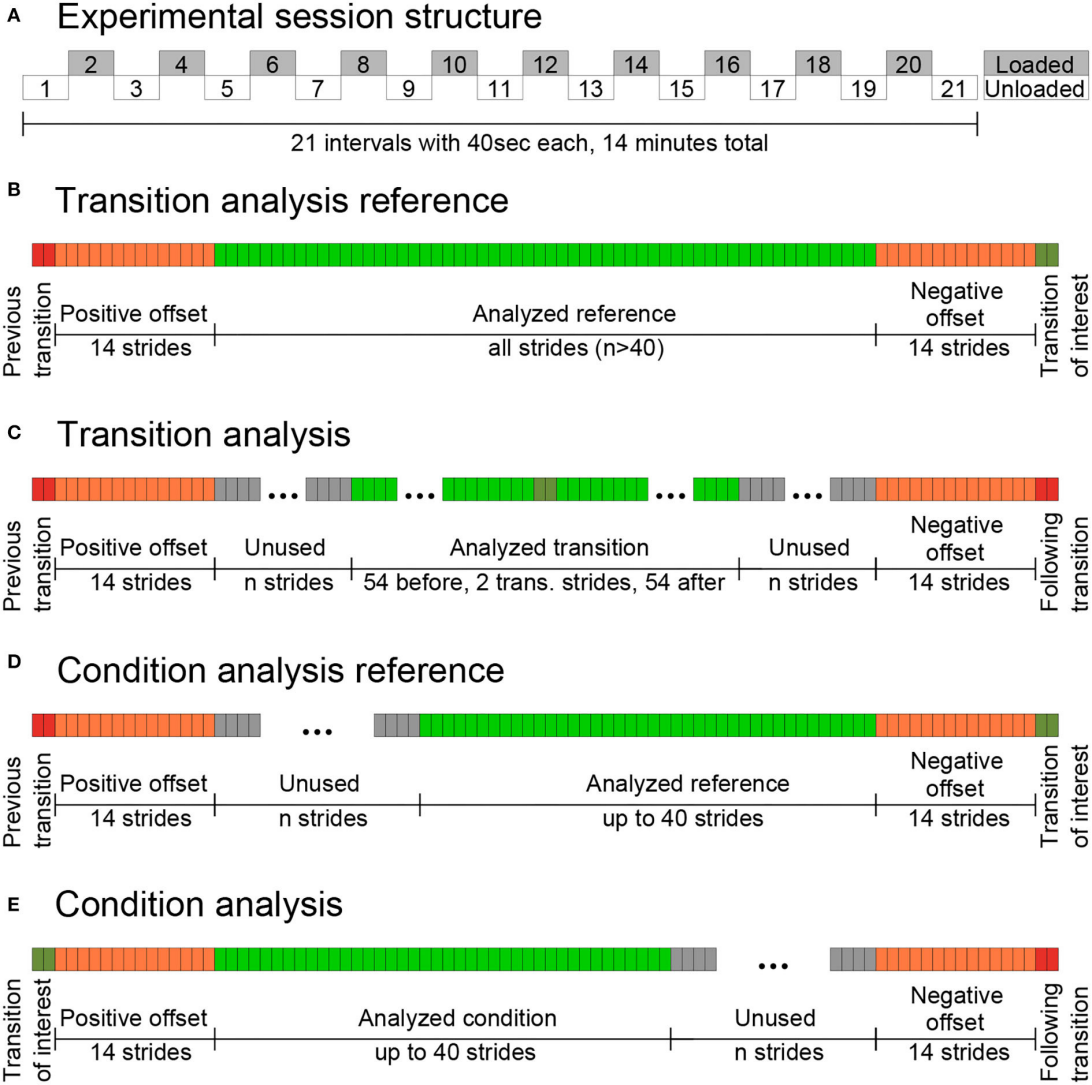
Experimental session structure for each weight session with 10 transitions to each loaded and unloaded walking **(A)**, stride selection for the transition reference **(B)** and the transition analysis **(C)**, and the stride selection for the condition reference **(D)** and the condition analysis **(E)**. Following Warmup session 1, sessions 2–4 included 21 intervals of alternating the loaded and unloaded conditions. For the 20 weight transitions, we first performed a transition analysis to identify and remove the transition strides, and then a condition analysis to evaluate the usability of the EMG from steady gait strides for HITL optimization.

Three weight sessions were performed in a balanced and randomized order, where subjects had to carry a 1, 2, or 4 kg weight in each hand, resulting in a total carried mass of 2, 4, and 8 kg for each of the weight sessions, respectively. After starting walking without holding any weights, every 40 s (during walking) subjects were instructed by an acoustic metronome to either pick up or release one pair of weights from/to a plate in front of the treadmill located at the height of the navel. In total, 21 intervals of 40 s were performed over 14 min, including 10 repetitions for each pick up and release of the weights, in each of the three weight sessions (Figure 3A). Subjects were instructed to always walk within arm's reach of the plate to maintain a constant position on the treadmill.

### 2.4. Data processing

The EMG and gyroscope data were exported to Matlab (Mathworks, Natick, MA, US) for further processing (Figure 2). Gyroscope data was interpolated to match the frequency of the EMG. Based on the individual signal timestamp from the Trigno Avanti data export, EMG and gyroscope data were synchronized.

#### 2.4.1. Gyroscope-weight transition identification

A method was required to detect the timing of the weight transition, as trial-to-trial variation was expected during transitions after hearing the metronome. A gyroscope was therefore affixed to each weight to record the angular velocity of the weights in three axes. The sum of the absolute value from all three axes was determined. In addition, this sum was filtered with a moving average having a sliding window of half of the EMG frequency. Following this, the sum of the left and right angular velocities was totaled. If the weights are not moving, the combined signal is nearly zero. When walking, the summed gyroscope signal is above 150°/s with larger peaks when picking up or releasing the weight. If a threshold of 40°/s is passed (in either direction), the corresponding left and right strides are considered to be transition strides.

#### 2.4.2. Gyroscope-stride identification

**Figure 4:**
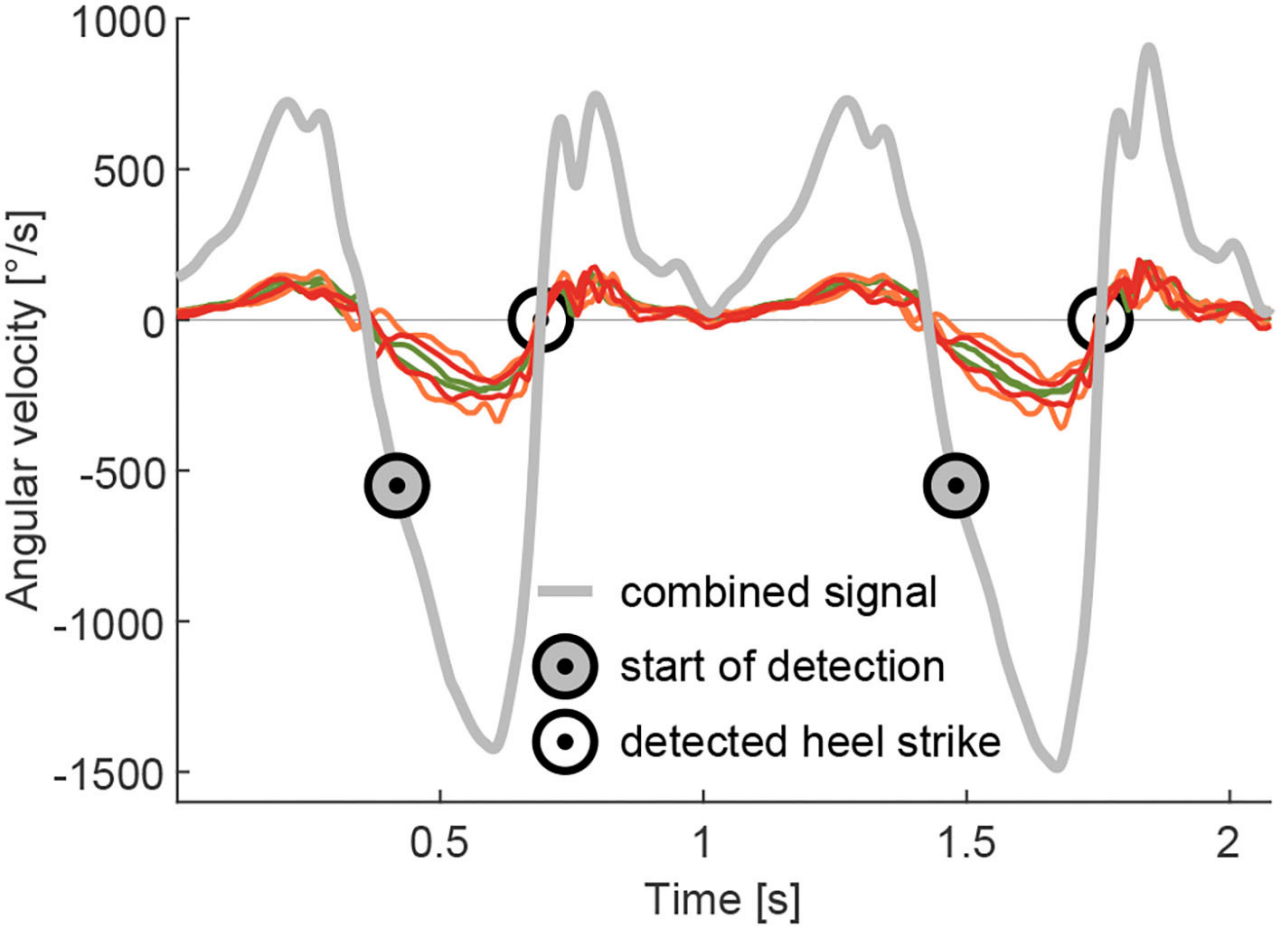
Example for the combined signal of the angular velocity during walking. The combined signal is the sum of the two axes, which include the movement in the sagittal plane, of the gyroscopes, integrated into the GAS (organe), SOL (red), and TIB (green) sensors. The combined signal was used for the detection of the heel strike to separate individual strides. The heel strike detection starts at an angular velocity of –550 °/s and identifies the following passing of zero, which is used as a heel strike event.

The stride identification (heel strike to heel strike of the same limb) is required to separate the EMG data of individual strides. This was accomplished based on the zero crossings of the lower limb segment angular velocities (Grimmer et al., 2016). In contrast to Grimmer et al. (2016) where a single gyroscope on the shank was used, a combined angular velocity signal of the sagittal plane of the TIB, GAS and SOL 3D gyroscopes was used (Figure 4). Due to the placement on the muscles, none of the three gyroscope axes of the TIB, GAS, and SOL sensors were perfectly aligned with the sagittal plane. The combined angular velocity was calculated as the sum of the two primarily involved axes of each sensor that recorded the angular velocity in the sagittal plane. As the EMG sensors with gyroscopes were not placed on rigid elements, we believe that the combination of the signals is more resistant to signal noise (e.g., oscillating soft tissue). Prior to identification, the combined signal data were low-pass filtered (zero-lag, 20 Hz, second-order Butterworth). As it was not required for our analysis, no adaptation was performed to account for the offset of zero crossings and the real heel strike. The stride time was calculated by subtracting the timestamp of the previous heel strike from the subsequent heel strike of the same limb.

#### 2.4.3. EMG-basic processing

**Figure 5:**
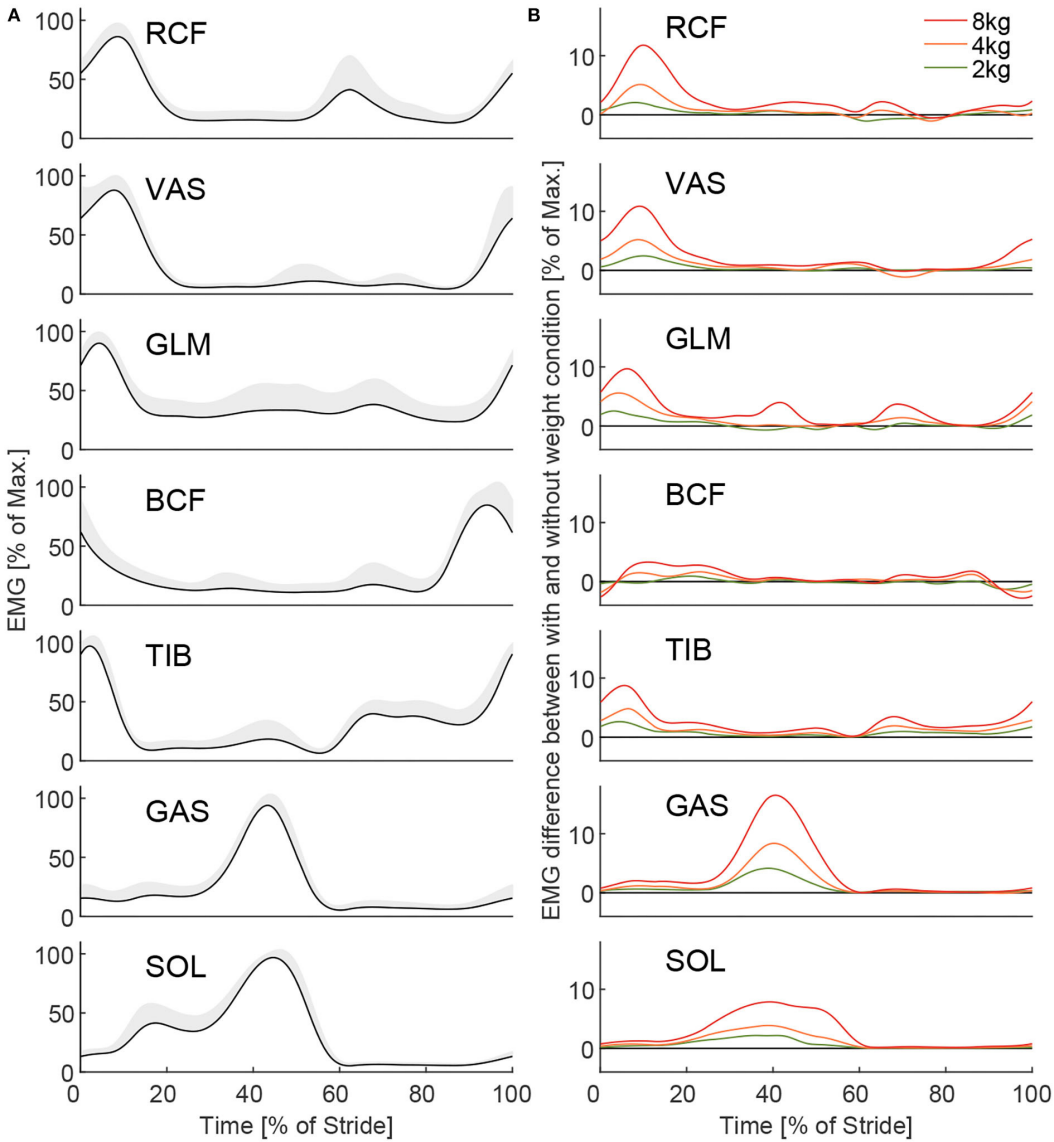
The Grand mean of the EMG for all muscles [**(A)**, left and right limbs combined] and EMG difference between the loaded and unloaded conditions **(B)** for 2, 4, and 8 kg. The grand mean is the mean of all subjects, strides, and intervals of the unloaded conditions from all three experimental sessions after the Warmup. Hundred percent represents the subjects' individual muscle maximum necessary for unloaded walking on average within the first interval of the first session after the Warmup. The gray shaded area indicates the inter-subject SD. Differences **(B)** between the loaded and unloaded conditions were determined based on the means (all intervals) of each condition for the same session. To note, due to a low signal-to-noise ratio some muscles [**(A)**, e.g., GLM] have a larger offset from zero, which does not necessarily indicate activation.

For all EMG signals, the offset was removed by subtracting the trial mean. Next, the EMG data was bandpass filtered (zero-lag, 40–450 Hz, fourth-order Butterworth) and rectified. Only for the time-normalized EMG over one stride and its differences between the loaded and unloaded conditions (Figure 5) were EMG signals additionally low-pass filtered (zero-lag, 6 Hz, second-order Butterworth).

As we realized that several EMG signals showed clear measurement artifacts, four strategies were applied to remove them. It should be noted that all strides that partially contained artifacts remained within the dataset to avoid temporal displacements but were excluded (replaced by NaN in Matlab) from each of the analyses.

First, phases with distinct changes in the EMG signal (e.g., to nearly zero signal or multiple times the previous signal) that happened within the 14 min sessions and that lasted for longer periods (shortest 12 s, on average 360 s) were completely excluded. For each subject, on average 5 of 14 muscles showed at least at one point during the four sessions a phase with a distinct change. With this method, about 6.5% of the data were excluded in total.

Second, distinct EMG peaks including 1 s prior to and following the peaks were excluded. Distinct peaks had more than three times the signal amplitude compared to the regular EMG peaks (mean peak height over the session) during walking. On average, about one error peak occurred per muscle within each 14 min session.

Third, next to distinct long-term and short-term changes in amplitudes, artifacts with similar amplitudes as the expected EMG signals, but with changes in duration or frequency of occurrence, were identified. To reduce the number of such artifacts, we explored different low cutoff frequencies for the bandpass filter. Typically the use of 10–20 Hz is reported (Rodríguez-Tapia et al., 2020) although maximum values of 30 Hz have been used in applications (Rodríguez-Tapia et al., 2020). Based on visual observation of the influence on the artifacts, we applied a low cutoff frequency of 40 Hz.

Fourth, after applying the first three strategies to exclude artifacts, the EMG signals for each stride were extracted based on the gyroscope data and time-normalized to represent the EMG of each stride by 1,000 frames. In addition, the mean absolute value (MAV, also known as average rectified value or ARV) of the EMG for each stride was determined, as this is our main variable for the EMG analyses.

The MAV was also used to exclude artifacts with similar amplitudes but prolonged time durations. Strides with MAVs that were five times above the SD of the mean MAV for each individual muscle, subject, and session were excluded (663 excluded out of a total of 5.56 x 10^5^, or approximately 0.1%).

Following this, the identified timing from the weight transition analysis was used to separate the EMG data for the loaded and unloaded conditions for the 21 intervals.

After removing artifacts, amplitude normalization was performed for the time-normalized EMG for the 14 individual muscles by determining the mean of the time-normalized EMG from the first unloaded interval from the first weight session (Figure 3, interval 1 in A). Based on the outcomes of the transition analyses (described later), we did not include the first (positive offset) and the last seven strides (negative offset) for each limb of the 40 s interval. An amplitude normalization factor was determined, which, when multiplied by the subject's time-normalized mean EMG of the first interval, sets the maximum value during the stride to 100%. As a final step, all individual time-normalized EMG curves (from all sessions and intervals) were multiplied by the subject- and muscle-specific amplitude normalization factor (13 subjects with 14 muscles results in 182 individual normalization factors).

#### 2.4.4. EMG-gait cycle analysis

To analyze the EMG changes during the gait cycle of walking (Figure 5), subject means of the time-normalized EMG trajectories for the left and right limbs were determined. Data from the left and right limbs were then averaged. Subject means were then combined to determine the grand means for each muscle and weight condition. The corresponding inter-subject SD was calculated. To determine the differences in EMG between loaded and unloaded conditions, the subject means of the unloaded EMG were subtracted from the loaded EMG for each subject, weight, and muscle individually. Following this, grand means for the weights and muscles were determined.

#### 2.4.5. EMG-transition analysis

A transition analysis was performed to determine the number of transition strides (negative and positive offsets) that should not be considered for the loaded and unloaded conditions during the EMG data comparison. For this analysis, the grand mean of the MAVs (MAV_stride_) and the grand mean of the intra-subject SD of the MAVs (MAV_stridestd_) were used. After normalization of the MAVs with a reference (Figure 3, green strides in B), MAV_strideand_ MAV_stridestdwere_ determined for 110 strides (54 strides prior to transition, 2 transition strides, and 54 post transition strides, Figure 3C).

**Figure 6:**
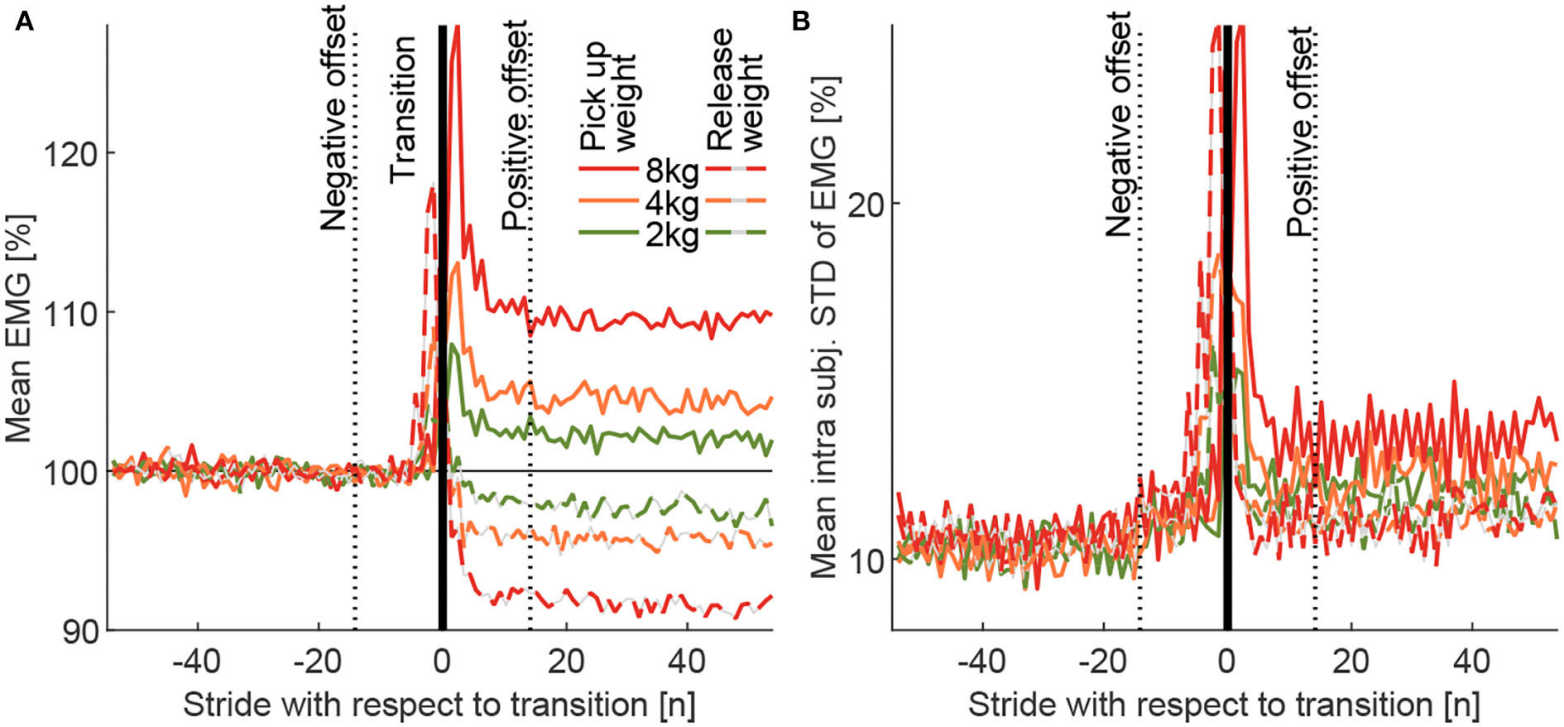
**(A)** The Grand mean of the MAVs (MAV_stride_) and **(B)** grand mean of the intra-subject standard deviation (MAV_stridestd_) for the transitions from the loaded to the unloaded condition (dashed) and the unloaded to loaded condition (solid) for the total carried mass of 2, 4, and 8 kg. The curves include data for all 5 muscles, both limbs (right, left), all 13 subjects, and all 10 transitions performed for each weight. The transition strides were defined by the movement of both weights. The follow-up condition is always presented relative to the previous condition, which is defined to have a mean of 100% (not including offset and transition strides). The positive (14 strides) and negative offsets (14 strides) were defined by visual observation of these curves.

The normalization with the reference was performed separately for the 110 MAVs of each subject, each of the 14 muscles, each of the 10 pick up and 10 release transitions, and each weight. MAVs were normalized to set the mean of all included MAVs of the analyzed reference strides (Figure 3, green strides in B) before the transition of interest to 100% (Figure 6A). All but the seven positive and seven negative offset strides (14 in total for both limbs) were included as reference strides (Figure 3, orange strides in B). It should be noted that the offset of seven strides per limb is based on the outcome of the transition analysis and was used in turn to improve the results.

Following this, the mean of the MAVs (over the 110 strides) of both the 10 pick up and the 10 release transitions were determined (for each weight, subject, and muscle). These means were used to determine the mean of all muscles (for each weight and subject). As a final step, these means were used to determine the MAV_stride_ based on all subject means (for each weight). Due to the normalization to the reference of 100%, the post-transition value of MAV_stride_ shows the relative change in EMG amplitude compared to the previous weight condition (e.g., 105% after the transition indicates an increase by 5%). The individual intra-subject SD was determined for the same 110 strides of each subject based on the 10 transitions to each weight condition for each of the three weights. The mean of this individual intra-subject SD was then determined for all muscles, and the grand mean MAV_stridestd_ was then determined by taking the mean of all subjects.

#### 2.4.6. EMG-condition analysis

After excluding the positive and negative offset and transition strides (transition process), the condition analysis was performed. The condition analysis includes up to 40 strides prior to and up to 40 strides post the transition process. The strides are indicated with *n*, where *n* ∈ [−40, −1] corresponds to the prior transition strides, and *n* ∈ [1, 40] corresponds to the post transition strides.

In the condition analysis, the MAVs were used to analyze whether it is possible to distinguish an increase or decrease in EMG after reaching steady walking following transitions between loaded and unloaded conditions. In contrast to the transition analysis, where each stride following the transition was compared separately to the whole reference condition prior to the transition, here the mean of the MAV_*n*_ of i consecutive strides (left and right combined), where *i* ∈ [1, 40] is investigated for both the reference prior and the comparison condition post the transition. MAV_*n*_ denotes the muscle MAV of the *n*th stride. Then the mean of the *i* strides prior to the transition MAV_*i*, prio_ is defined as


(1) $$\begin{array}{l} {\text{MAV}}_{i,\text{prio}}=\frac{1}{i}\overset{-1}{\underset{n=-i}{\sum }}{\text{MAV}}_{n}\text{.} \end{array}$$

The mean of the *i* strides post to the transition MAV_*i*, post_ is defined as


(2) $$\begin{array}{l} {\text{MAV}}_{i,\text{post}}=\frac{1}{i}\overset{i}{\underset{n=1}{\sum }}{\text{MAV}}_{n}\text{.} \end{array}$$

The consecutive prior (reference) and post (comparison) transition strides always start close to the transition of interest. For example, for the case of the 10 stride combination (*i* = 10, five left and five right consecutive strides) the mean of the last 10 strides before the transition of interest (MAV_10, prio_, reference condition, Figure 3D) and the mean of the first 10 strides after this transition (MAV_10, post_, comparison condition, Figure 3E) are compared. The comparisons were separately performed for each subject, for each of the seven muscles from the combined data for the left and right limbs, and each weight. For each comparison, the MAVs of the included strides were normalized to achieve a mean of 100% for the MAV_*i*, prio_ (for each limb separately). In the case of picking up the weight, an increase in muscle activity would be expected, which would result in a normalized MAV_*i*, post_ of larger than 100%. In the case of releasing the weight, the opposite would be expected to occur.

At this stage, a data structure exists, which contains 13 subjects, each with three different weights, and each containing seven muscles (left and right combined). Each muscle contains a matrix with 20 x 40 values that includes the MAV_*i*, post_ for 20 transitions (10 pick up, 10 release) and the stride combinations for one to 40 strides.

A decision must be made regarding which of the seven muscles or which combination of muscles should be analyzed. We decided to analyze all possible 127 muscle combinations including either one (7 possible combinations), two (21), three (35), four (35), five (21), six (7), or seven (1) muscles. For combinations of muscles, the means of all values of MAV_*i*, post_ from the included muscles were determined. For example, for a combination of two muscles where one had a MAV_*i*, post_ of 103% and the other of 107%, the combined mean MAV_*i*, post_ would be 105%.

Based on 10 transitions and 13 subjects for each the pick up and the release of weight, in the best case without any exclusions due to the artifact removal, the total number of evaluated transitions *K* equals 130. Let *k* ∈ [1, *K*] indicate the *k*th measurements. Following this, from all subjects and transitions, we counted the number of changes where the change of muscle activity occurred in the expected direction (*E*_*k*_).

Then MAV_*i*, prio, *k*_ and MAV_*i*, post, *k*_ denote the prior and post transition MAVs of the *k*th measurement.

For a pick up of weight


(3) $$\begin{array}{l} E_{k}=\left\{ \begin{array}{cc} 1, & \text{if }{\text{MAV}}_{i,\text{prio},k}<{\text{MAV}}_{i,\text{post},k}\\ 0, & \text{otherwise} \end{array}\right. \end{array}$$

is 1 if the MAV_*i*, post_ of the *k*th transition increases as expected, and 0 if it unexpectedly decreases also while picking up a load. In case of releasing of weight


(4) $$\begin{array}{l} E_{k}=\left\{ \begin{array}{cc} 1, & \text{if }{\text{MAV}}_{i,\text{prio},k}>{\text{MAV}}_{i,\text{post},k}\\ 0, & \text{otherwise} \end{array}\right. \end{array}$$

is 1 if the MAV_*i*, post_ of the *k*th transition decreases as expected, and 0 if it unexpectedly increases also while releasing a load. Then


(5) $$\begin{array}{l} K_{\text{E}}=\overset{K}{\underset{k=1}{\sum }}E_{k} \end{array}$$

is the number of transitions that behave as expected for each pick up and release. The detection rate is defined as


(6) $$\begin{array}{l} \text{DR}=\frac{K_{\text{E}}}{K}\;. \end{array}$$

and is calculated for all 127 muscle combinations and the three weights.

As we can not present the results for all 127 muscle combinations for both transitions and for each MAV_*i*, post_ we focused our detailed analysis on specific questions.

**Figure 7:**
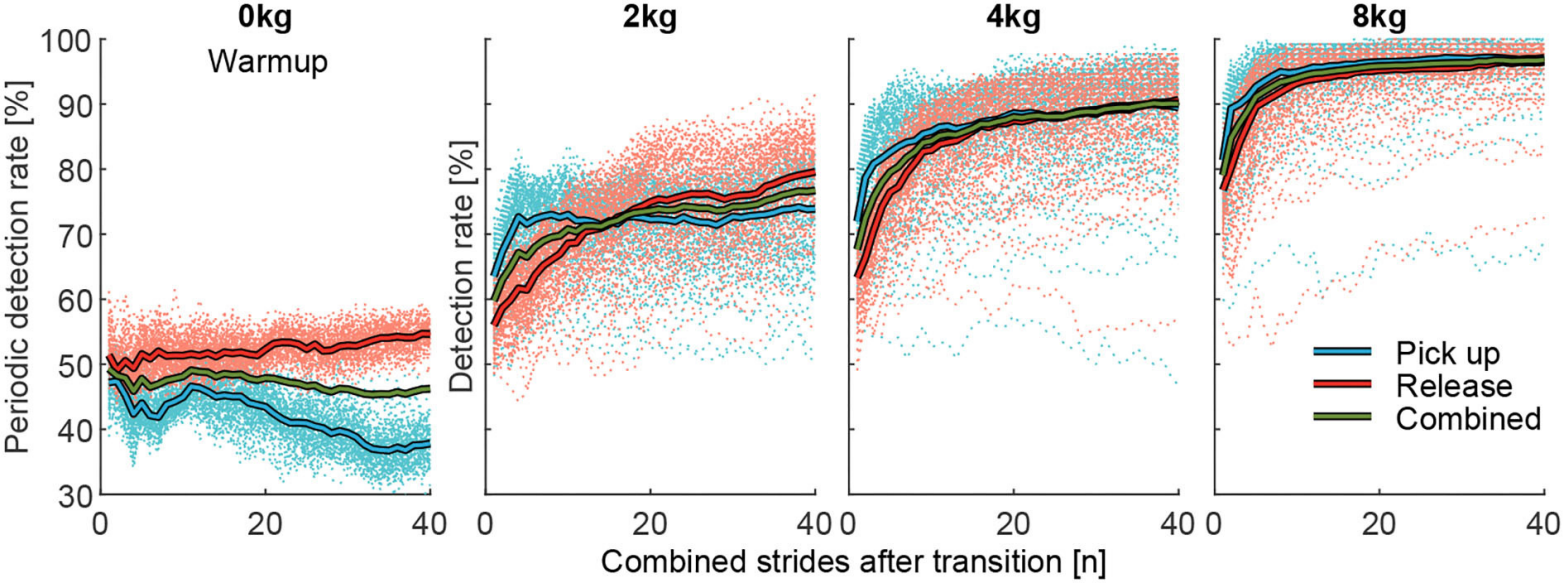
Detection rate (DR) of the periodic analysis (Warmup, 0 kg) and the actual (2, 4, and 8 kg) changes in effort for picking up (red) and releasing (blue) the weight, and the combined value (green). Thick solid lines indicate the mean of the respective 127 individuals (dashed lines) muscle combinations. While the Warmup only contains unloaded steady walking, for the periodic analysis, a combination of up to 40 strides was included based on the timing of the 21 intervals (40 s each) to show the effect of EMG drift on the DR for a change in walking effort.

To identify differences in the pick up and the release behavior for DR, the grand mean (including all subjects and transitions) of all 127 muscle combinations was determined for each transition case (pick up and release) separately (Figure 7). Additionally, a combined (mean) grand mean of the pick up and release condition was determined. Based on the 40-s intervals, we also performed this procedure for the Warmup where no weight was picked up or released (indicated as periodic analysis) to identify how EMG drift influences DR.

**Figure 8:**
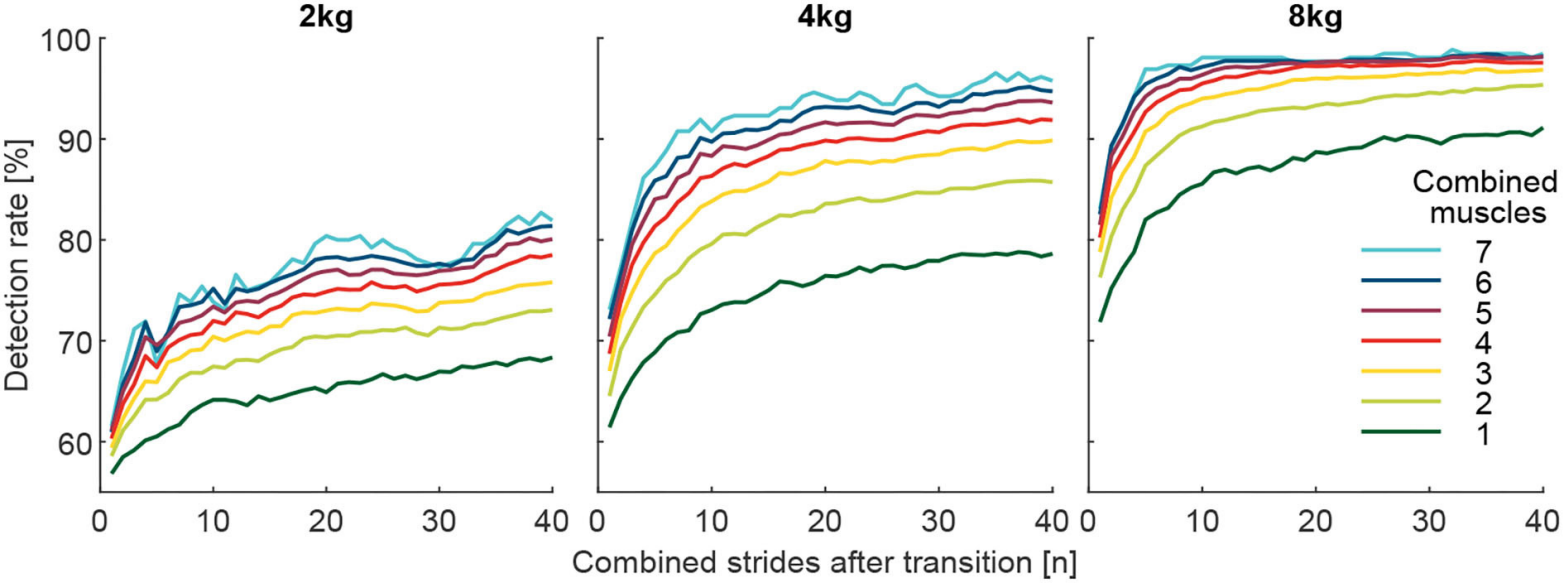
Detection rate with respect to the number of involved muscles from the 127 possible muscle combinations analyzed. Each graph includes 40 strides in alternating order from the left (20 strides) and right (20 strides) limbs. For example, the dark green curve is the mean of the seven individual muscle analyses, and the light green is the mean of the 21 possible two-muscles combinations. Furthermore, all combinations with 3 (yellow, 35 combinations), 4 (red, 35 combinations), 5 (purple, 21 combinations), 6 (blue, 7 combinations), and 7 (cyan, 1 combinations) involved muscles were analyzed.

To investigate whether the identification of the weight change can be improved by increasing the number of involved muscles (Figure 8), we calculated grand means (including all subjects and transitions) of DR (for *i* involved strides after the transition) based on the number of involved muscles. For example, in the case of two-muscle combinations, the mean of 21 combinations was determined. For this analysis, the values for the pick up and release were combined (mean).

**Figure 9:**
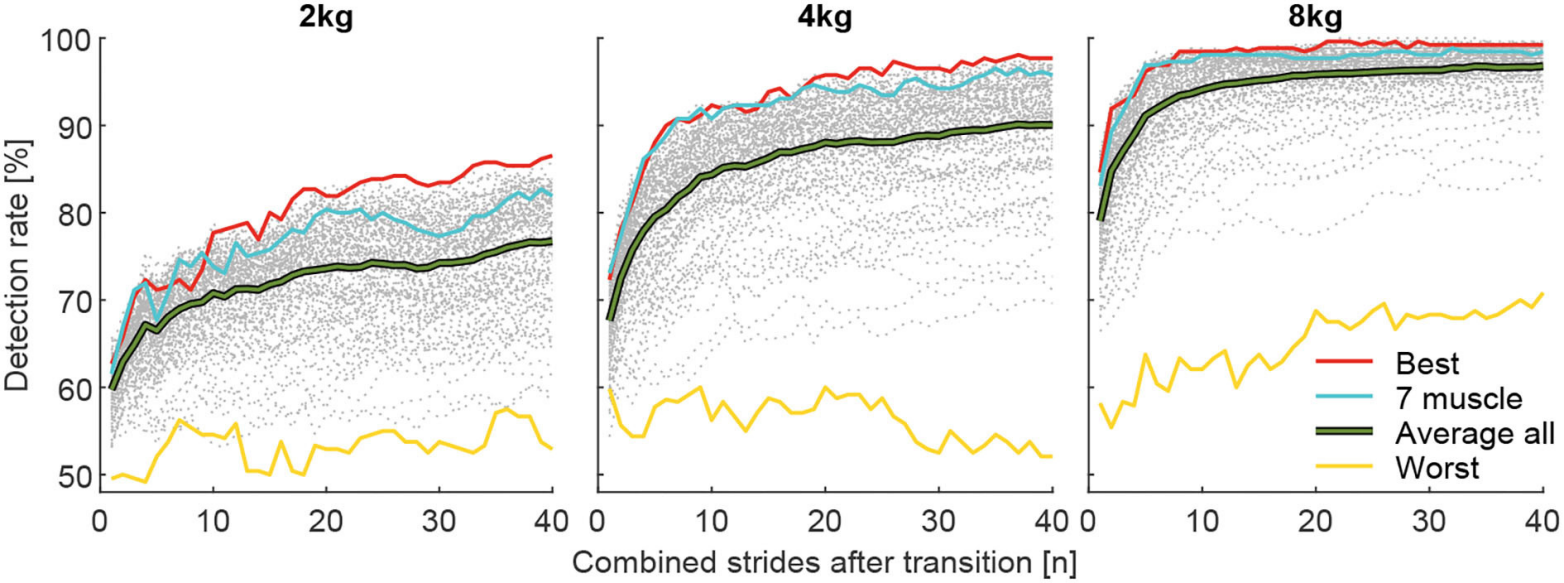
Detection rates for the total carried mass of 2, 4, and 8 kg for the best muscle combination (red), the seven muscle combinations (blue), the average of all combinations (green), and the worst combination (yellow). Each graph includes 40 strides in alternating order from the left (20 strides) and right (20 strides) limbs.

**Figure 10:**
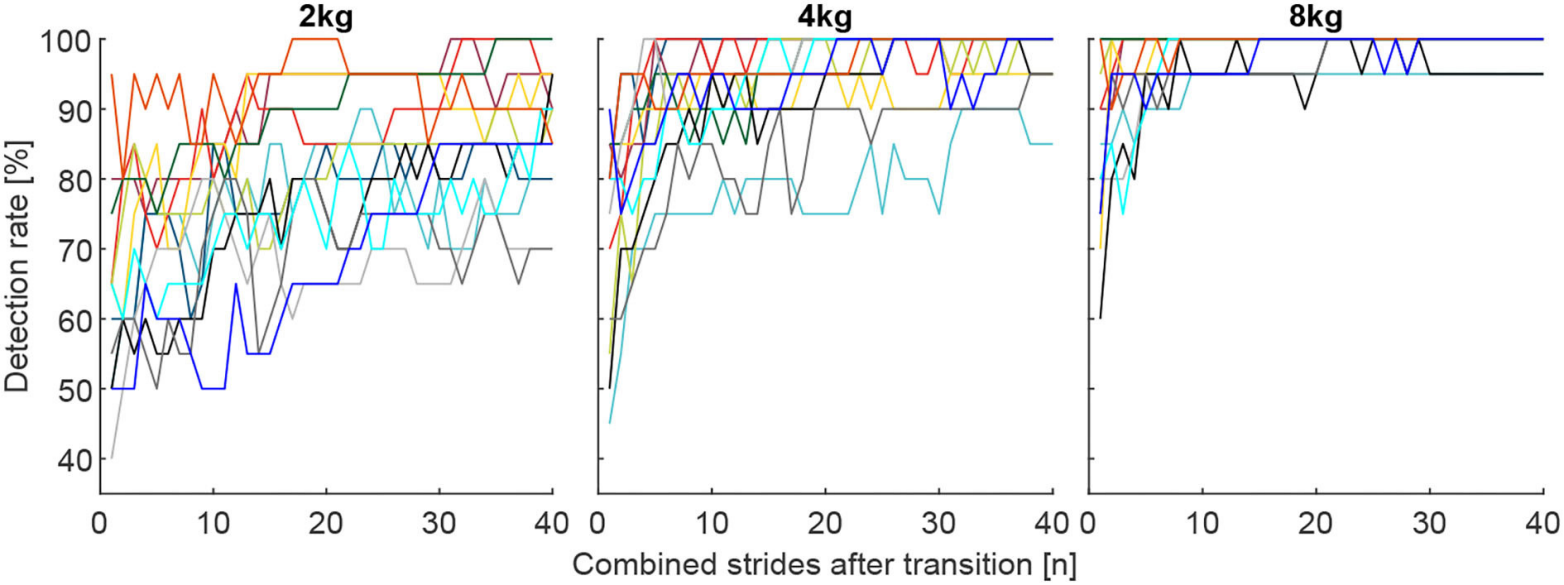
Individual detection rates for the 13 subjects (each color represents one subject) for the total carried mass of 2, 4, and 8 kg for the best muscle combination, which includes the VAS, SOL, GAS, and GLM for the 2 kg and 4 kg conditions and the VAS, SOL, GAS, RCF, and TIB for the 8 kg condition. Each graph includes 40 strides in alternating order from the left (20 strides) and right (20 strides) limbs.

To identify the best and worst possible DR and the associated muscle combination, the combinations with the highest (Best) and lowest (Worst) grand mean (including all subjects and transitions) of all DR for *i* involved strides were determined (Figure 9). To show the variability in between subjects, DR of the individual 13 subjects for the muscle combined with the highest grand mean is shown with respect to *i* involved strides in Figure 10. For both analysis, the values for the pick up and release are combined (mean).

**Figure 11:**
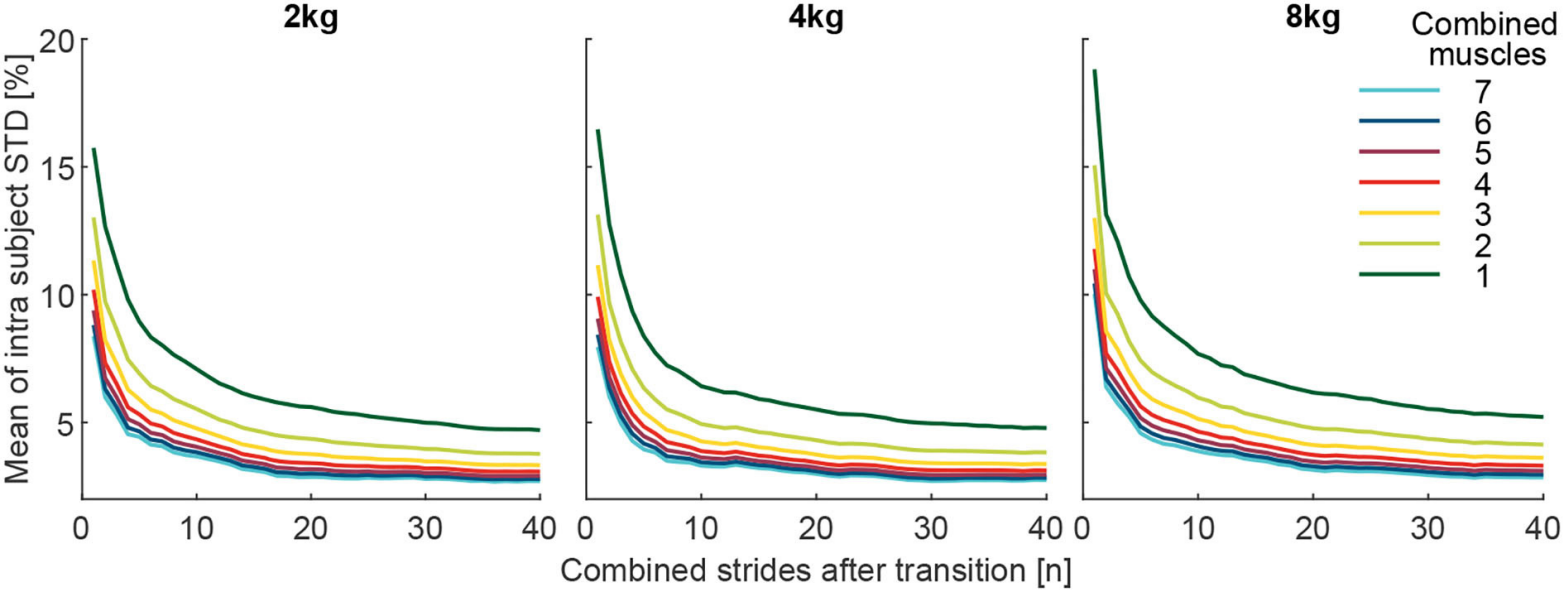
The Grand mean of the intra-subject SD (MAV_*i*, std_) of the effort level (MAV_stride_) of the comparison condition (above or below the reference of 100%, depending on the condition). The MAV_*i*, std_ is shown with respect to the number of involved muscles from the 127 possible muscle combinations analyzed. Each graph includes 40 strides in alternating order from the left (20 strides) and right (20 strides) limbs. For example, the dark green curve is the mean of the seven individual muscle analyses, and the light green is the mean of the 21 possible two-muscle combinations. Furthermore, all combinations with 3 (yellow, 35 comb.), 4 (red, 35 comb.), 5 (purple, 21 comb.), 6 (blue, 7 comb.), and 7 (cyan, 1 comb.) involved muscles were analyzed.

To better understand the reason behind the changes in the detection rate with an increased number of muscles and strides, the grand mean of the intra-subject STD MAV_*i*, std_ of the MAV_*i*, post_ was determined. To obtain the MAV_*i*, std_, for each subject and each muscle the STD of the MAV_*i*, post_ of both the 10 pick up and 10 release transitions were determined. Following this, the STD was averaged over all muscles and then over all subjects. For the results presented in Figure 11, the mean of all respective muscle combinations (including one to seven muscles) for the MAV_*i*, std_ was determined.

#### 2.4.7. EMG-drift analysis

**Figure 12:**
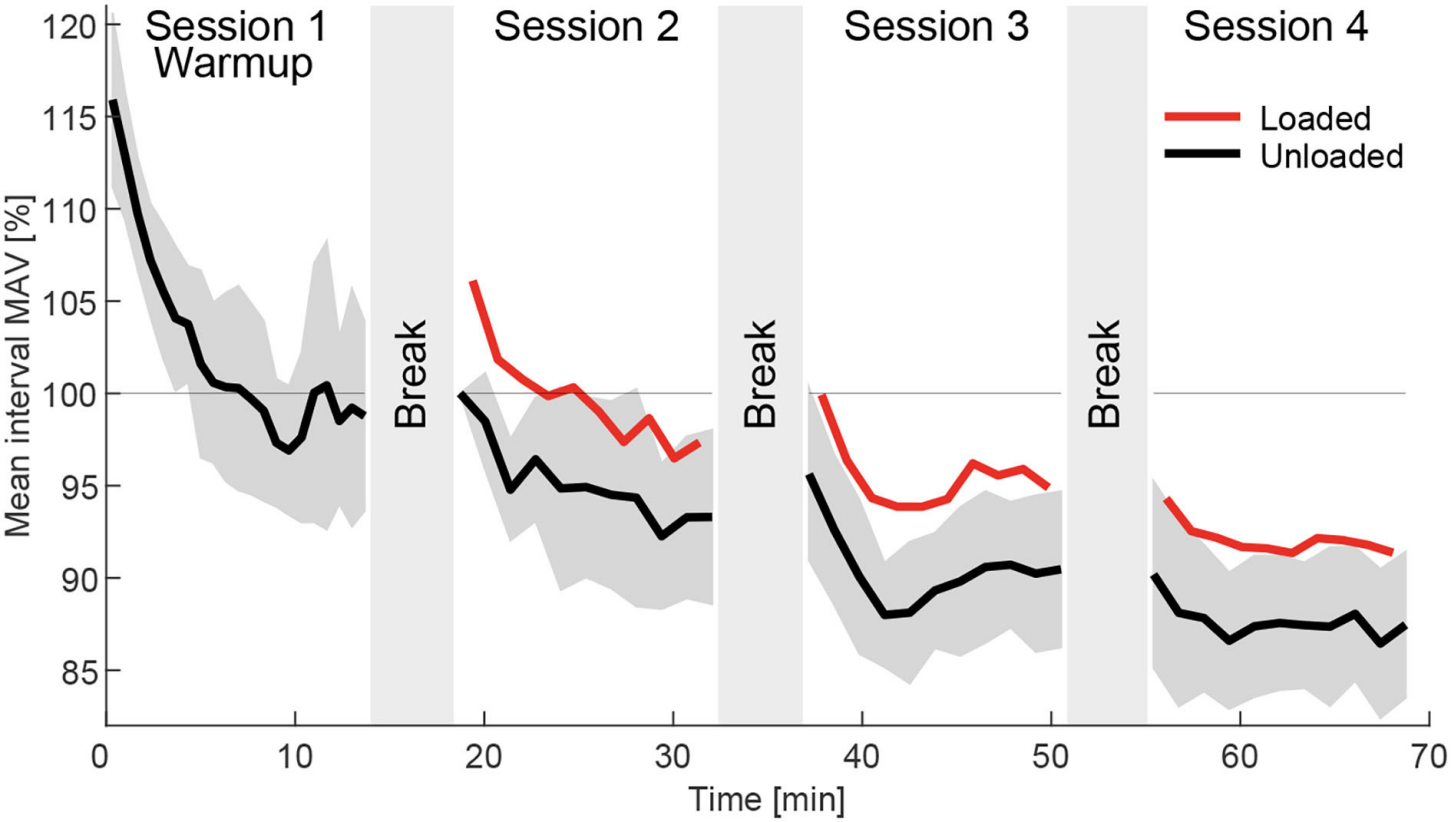
Grand mean interval MAV (MAV_int_) for the loaded (red) and unloaded (black) condition in the order they were performed during the experiment, which included four sessions beginning with the Warmup followed by the three weight sessions in randomized order. The data includes the strides of all subjects and muscles of both limbs without transition and offset strides. The data was normalized to the MAV_int_ of the first interval of session 2. The standard deviation of the 14 muscles of the unloaded data MAV_intstd_ is indicated in dark gray. Average break times in between sessions were determined and indicated by the gray areas in between sessions. A clear negative drift of the MAV_int_ was found over time.

To identify how the MAV drifts over time during walking (Figure 12), instead of analyzing the three weights, the analysis was performed based on the temporal order of the four experimental sessions. For each of the four sessions and each intervals a grand mean session interval MAV (MAV_int_) was separately determined from the loaded and unloaded conditions. To compute MAV_int_, all but the positive and negative offset MAVs and the two transition MAVs were averaged for each interval and all subjects. Similar to the normalization approach explained previously, these values were normalized to be 100% for the first interval (unloaded) of the first session after Warmup. These values were then used and averaged over all 14 muscles to determine MAV_int_ and the related SD between the muscles MAV_intstd_.

## 3. Results

### 3.1. Gait cycle analysis

When walking for approximately 40 s at 1.3 m/s for each interval, subjects took an average of 65 strides for the left and right limbs combined. Stride time did not change considerably for the different weights and conditions (Table 1).

**Table 1:** Stride time [s] for the Warmup and the different weights and their loaded and unloaded conditions.

**Condition**	**Warmup**	**2 kg**	**4 kg**	**8 kg**
Loaded	–	1.10 ± 0.04	1.10 ± 0.05	1.10 ± 0.05
Unloaded	1.09 ± 0.05	1.09 ± 0.05	1.10 ± 0.05	1.09 ± 0.05

With unloaded walking, we found EMG values (Figure 5A) to be comparable to other published data (Hof et al., 2002; Den Otter et al., 2004). Clear increases in muscle activity between the loaded conditions and the unloaded conditions were identified for all muscles except for BCF (Figure 5B). The peak increases occur during phases of typical peak muscle activity in unloaded walking, they scale with carried weight, and they reach the highest values for the weight of 8 kg (increase of up to 17%, Figure 5B). Mean EMG increases (based on strides 16–55 of MAV_stride_) of 2.1, 4.4, and 9.5% were found when picking up the weight, and –2.4, –4.3, and –8.4% were found when releasing the weight for 2, 4, and 8 kg, respectively (Figure 6A).

The average grand mean of the intra-subject SD MAV_stridestd_ of the EMG was found to be (based on strides 16 to 55) 12.1, 12.3, and 13.5% for picking up, and 11.3, 11.1, and 11.1% when releasing the weight of 2, 4, and 8 kg, respectively (Figure 6B).

### 3.2. Transition analysis

To exclude the strides of the transitions from the EMG comparison of the loaded and unloaded conditions, the transition strides and the enclosing offset strides with non-steady-state gait were identified. Based on visual inspection of MAV_stride_ and MAV_stridestd_ (Figure 6), we found 14 negative offset strides, followed by the two transition strides (left and right) and 14 positive offset strides.

### 3.3. Condition analysis

When analyzing the grand mean of DR for the pick up and release conditions (Figure 7), we found that when picking up a weight DR increases faster and settles earlier (especially for 2 kg) with an increased number of involved strides compared to when releasing a weight. Additionally, in the periodic analysis without a weight change during the Warmup, DR increases slightly for the release and decreases slightly for the pick up condition.

When analyzing DR of all possible 127 combinations of the involved seven muscles, we found that combinations that include more muscles provide, on average, a higher detection rate for the change in walking effort when comparing the loaded and unloaded conditions (Figure 8). Furthermore, we found that using mean EMG values of multiple strides for the reference (before transition) and the comparison condition (after transition) helps to increase the detection rate (Figures 8, 9).

On average, the use of seven muscles of both limbs, instead of just one, improved DR by 12, 17.4, and 10.2% for the 2, 4, and 8 kg conditions, respectively (Figure 8).

The muscle combinations with the highest DR (Figure 9) for each weight always included the VAS, SOL, and GAS muscles. The GLM was also included for the 2 and 4 kg conditions, and the RCF and TIB were also included for the 8 kg condition. For each weight, the worst combination includes only the BCF.

The corresponding highest DR after one stride were 62.7, 72.3, and 84.6%, after ten strides 77.7, 92.3, and 98.5% and after 40 strides 86.5, 97.7, and 99.2% for the 2, 4, and 8 kg conditions, respectively.

Individual subject data (for the combination of muscles found to result in the highest DR for the overall population) reveals a minimum DR of 50% for a single subject after 10 strides, 65% for two subjects after 20 strides, and 70% for two subjects after 40 strides (Figure 10).

The grand mean of the intra-subject SD (MAV_*i*, std_) was found to decrease greatly with an increased number of combined strides and increased number of muscles for all weights (Figure 11).

### 3.4. Drift analysis

When analyzing the grand mean interval MAV (MAV_int_) during the course of the whole experiment for the loaded and unloaded conditions, we found a clear negative drift (Figure 12). Drift rates were largest at the beginning of each session and generally became smaller as the session went on. From session 1 to session 4 MAV_int_ changed from 116% at the beginning of session 1 (Warmup), to 100% at the beginning of session 2, and to 88% at the end of session 4. Similar drift was found for the loaded condition where MAV_int_ dropped with respect to the unloaded condition from 106% at the beginning of session 2 to 91% at the end of session 4.

## 4. Discussion

### 4.1. Transition analysis

When changing the weight condition, subjects require a certain number of strides to acclimatize to the new pattern. So far, it is unknown to what extent the EMG of these transition strides can be used for HITL optimization. We decided to exclude these strides from our analysis because, when changing weight, the MAVs of the transition strides were larger and had a larger intra-subject STD compared to the previous and following steady walking strides. Based on visual observation, 30 strides of both limbs combined were excluded, including 14 strides prior to the defined transition strides, one transition stride for each limb, and 14 strides following the transition strides. While being very conservative with our exclusion procedure, excluding 30 strides was slightly more than expected.

However, we believe that transitions between assistance patterns for lower limb robotics can be realized in fewer strides, as for our scenario the subjects had to additionally accomplish the tasks of picking up or the release of weight. Furthermore, we could not perfectly identify the beginning of the transition to synchronize all transitions based on the first transition stride, as the selected event for the transition identification (weight movement) was within the process. As the acoustic metronome was not perfectly synchronized with the EMG and subjects reacted at different times after hearing the metronome, we did not use the metronome as the event to evaluate the duration of the transition. Additionally, as it was not the main focus of our study, we decided to use one duration for all conditions even though the negative or positive offset could be reduced for individual subjects, amount of weight, the pick up condition, or the release conditions.

Literature shows that transitions where the environment forces a transition, such as a transfer from level walking to stair ambulation, are realized within three strides (Grimmer et al., 2020). Three strides are also required to return to a steady gait after stumbling perturbations (Cordero et al., 2003). We can imagine that more than three strides are necessary to optimize muscle behavior for an unknown assistance pattern. In Yokoyama et al. (2018), the adaptation process for a change in speed of a single belt of a split-belt treadmill was explored. In addition to an immediate response to the new belt speed, variables were found that showed further changes over the next 5 min. While the instantaneous adaptation was attributed to reactive control, the longer adaptation process was attributed to predictive control. Long-term adaptation (training) effects were also demonstrated when performing training with an exoskeleton over longer periods of time and over multiple days and sessions (Koller et al., 2015; Panizzolo et al., 2019).

Based on previous findings and our results, we believe that it should be sufficient to exclude between 3 and 20 strides to capture the main biomechanical adaptation to a new assistance pattern, which translates to removing a maximum of 11 s of data for each transition. However, the subsequent strides of the removed strides will never represent the same walking biomechanics and level of effort as when acclimatizing to the new assistance pattern for several minutes over multiple sessions. It is unclear to what extent this adaptation influences the quality of HITL optimization.

### 4.2. Condition analysis-multiple consecutive strides

The combination of the same number of consecutive reference and comparison strides (MAVs) was, as expected, highly beneficial for DR, where the largest increases were found for the first few added strides. For the best muscle combination, increases of 15 and 20% were found when averaging over 10 strides while 30 strides simply improved DR by another 9 and 5% for the 2 kg and 4 kg conditions, respectively. The DR with 8 kg weight settled to nearly 100% after 10 strides. Thus, based on our study, we would advise using at least 10 strides (about 5.5 s) for HITL optimization. Similar values were identified in Shiavi et al. (1998). However, our minimum weight was 2 kg and there will likely be a lower DR for conditions that result in a lower change in human effort. In addition, we found that individual subject data could be insufficient to distinguish between conditions with different walking efforts after 10 strides (Figure 10). Therefore, it may make sense to perform a rough search for assistance patterns within the solution space with 10 strides and then to increase the sensitivity with the help of an increased number of strides. As DR for the 2 kg condition did not settle after 40 strides, it may also make sense to include the strides beyond 40.

### 4.3. Condition analysis-multiple muscles

As expected, with an increased number of included muscles DR increased. For all three weights, the seven-muscle combination was close to the best achieved performance (Figure 9). Our data also revealed that combinations of fewer muscles can achieve maximum or close to maximum performance. For example, for the 4 kg weight, the combination of VAS, GLM, SOL, and GAS achieved the highest detection rate.

However, as there is a variety of possible manipulation scenarios for lower limb robotics including the hip, knee, and ankle, or combinations of these joints, it will be a challenge to select those muscles that are primarily affected. For example, when assisting the hip also the EMG of the SOL and GAS changed (Franks et al., 2021). In addition, perturbing the lower limb could also lead to increased demands to maintain a stable gait by lower limb placement, trunk movements, or arm movements (Hill and Nantel, 2019). While one EMG signal may decrease others could increase, and this makes it challenging to predict the overall outcome of the movement effort. In our data, the BCF showed almost no increases in activity for the increased weight levels (Figure 5B). Even a negative effect was identified in the late swing. As a result, the only BCF muscle condition performed the worst when detecting the transition between the loaded and unloaded conditions (Figure 9). Thus, we would recommend involving as many muscles as possible for scenarios where the effect on the EMG is unknown. In known scenarios, the number of involved EMG signals could be reduced to the ones with the highest impact (Han et al., 2021). In addition, it has to be investigated how using an exoskeleton impacts the EMG and effort to stabilize gait with lower limb, trunk, or arm movements. For extreme experimental scenarios in running (without exoskeleton), it was found that without arm swing net metabolic cost increased by 7.6%, and with external lateral waist stabilization net metabolic cost decreased by 12.3% (Arellano and Kram, 2012).

### The reasoning for the benefits of multiple muscles and strides

We found that a major reason for the positive effect on detection rates is the reduced variability of the MAVs when averaging over a larger number of strides and/or larger number of muscles for both the reference and the comparison condition. The variability was found to decrease for an increased number of strides and muscles (Figures 8, 11).

Assuming the single-stride MAVs of the EMG are normally distributed with SD σ (for consecutive strides or multiple muscles), the sample mean will also be normally distributed with the SD. The sample SD $\frac{\sigma }{\sqrt{n}}$ decreases with increasing the number of samples *n*. Hence, by increasing *n* (the number of strides or muscles) the distribution of the estimator will be more narrow and the distinction between loaded and unloaded conditions will improve. However, with every increase of *n*, for either strides or muscles, the level of DR improvement decreases (Figure 8). For muscles, the improvements are small beyond five muscles, and for strides, the improvements are small beyond ten strides for the 8 kg condition and 20 strides for the 2 kg condition.

These findings suggest that the reduction in EMG variability due to biological and non-biological sources is key to improving the usability of HITL optimization. Thus, future studies should explore methods to reduce EMG variability. In addition, other time and frequency domain EMG features (Nazmi et al., 2016) could be explored on their variability and suitability for HITL optimization. For example, the root mean square (RMS) was already used in some works performing EMG-based HITL optimization (Zhang et al., 2017; Han et al., 2021).

### 4.4. Drift analysis

We found that the MAV_int_ of the EMG has a negative drift over time (Figure 12). As expected, the drift was largest during the Warmup and was reduced within the following sessions. Furthermore, breaks from walking seem to remove part of the drift but lead to larger drift rates after starting the following session. Our study provides no clear answer for the origin of the drift but we believe it is a mixture of several sources.

When looking at individual muscle data, especially at the beginning of the Warmup, the EMG showed much larger amplitudes compared to the following sessions. We believe that these are artifacts due to the sensor and skin movements, which are reduced later in the experiment as sweating improves the conductivity between the sensor and the skin (Day, 2002).

While sweating initially helps to attain a stable signal, even at low intensity levels sweat can accumulate and become a confounding factor (Abdoli-Eramaki et al., 2012). Sweating was found to decrease EMG amplitudes for a maximum voluntary contraction by almost 50% for sweat layers of 0.2 mm (Abdoli-Eramaki et al., 2012). The authors assume that a decrease in skin resistance leads to an increase in skin conductivity between the electrodes of the sensor, which causes a short circuit for the signal. Sweat ion concentrations also seem to influence EMG (Takagi et al., 1962).

Skin temperatures have also been found to affect EMG amplitudes. Winkel and Jørgensen (1991) found that with ambient temperatures of 14 and 30° C, the skin temperature of the investigated muscles was found to be 21.7 and 32.9° C, respectively, and this was found to reduce the EMG amplitude by 50% for the higher temperature. Our experiments were performed at a comfortable room temperature, though we neither checked nor controlled for the room temperature within our measurements (about 70 min). No temperature changes were noted by either the subjects or the experimental team. However, as was previously found (Fröhlich et al., 2015), increases in skin temperature during the Warmup could explain the identified reductions in MAV_int_.

In addition to skin temperature, changes in the muscle temperature due to a Warmup (Stewart et al., 2003) can result in an internal optimization of muscle use, and this can lead to higher muscle force and power output with a reduction in muscle activity.

Temperature changes in the measurement system could be also a possible source of EMG changes (Takagi et al., 1962). However, as the EMG system was running for at least 30 min in advance of the experiments we do not expect considerable effects on our results.

Fatigue could be also a reason for the observed EMG drift (Barsotti et al., 2020; Eken et al., 2020). However, study outcomes on the relationship between fatigue level and EMG are ambiguous (Mizrahi et al., 1997; de Oliveira et al., 2017; Eken et al., 2020) and the sources for the identified changes in EMG (Eken et al., 2020) are unknown (Vøllestad, 1997) and may not necessarily be fatigue related.

Based on the outcomes of this study, we would recommend performing at least 6 min of Warmup before starting the HITL optimization with the MAV of the EMG as feedback, which is a similar duration as that recommended for stable biomechanical performance (Meyer et al., 2019). If EMG-based variables other than the MAV are used, we recommend also assessing them for similar drift. Furthermore, based on our findings, we recommend avoiding breaks to avoid large changes in the MAVs after the breaks. We also recommend only comparing the EMG outcomes from temporally adjacent or even consecutive assistance patterns rather than comparing outcomes from the beginning of an optimization to outcomes from the latest patterns during protocols lasting up to 1 h.

### 4.5. Methodological considerations

#### 4.5.1. Artifact removal

We used four methods to remove artifacts from the EMG data. Distinct long-term changes in amplitudes, distinct peaks, and low frequency artifacts leading to large MAVs were removed. We believe that all but the first method can easily be applied online. For the first method, a reference for an artifact-free EMG of each subject in combination with certain thresholds would be required for online use. To investigate the overall effect of the artifact removal, we also performed our analysis without using the four removal methods (lower cutoff frequency of the bandpass filter typically set to 10 Hz). The detection rate (Figure 9) and its STD (Figure 11) were impacted in a slight negative fashion. For the best combination, the detection rates dropped after 10 strides to 77.3, 90.8, and 96.9%, and after 40 strides to 80.4, 95, and 97.7% for 2, 4, and 8 kg, respectively.

In contrast, without the artifact removal, the drift of MAV_int_ over the whole experiment increased and covered a range from 134% at the beginning of the Warmup to 82% within session 4, a range of 52%, compared to 28% when using the artifact removal. Without artifact removal, the STD of MAV_int_ doubled.

One possible source of the artifacts could be the weight (14 g) of the new generation of mobile EMG sensors, which include additional electronics (e.g., IMU, battery). Human wobbling masses of the thigh and shank were found to move within 3–55 Hz following the impact during heel-toe running (Schmitt and Günther, 2011). We can imagine that these frequencies are also reflected in the noise of the EMG signal.

While the EMG artifact removal had only a minor positive effect on the detection rate, we would still recommend its use as implementation is easily possible online for most of the methods we have implemented.

#### 4.5.2. The lower cutoff frequency of the bandpass filter

One curiosity was explored within our evaluation. We suspected that lower cutoff frequencies of the bandpass filter above 10–20 Hz would considerably change the means of the EMG data (Figure 5). However, after normalizing the signals to the maximum activity within a stride (100%), the changes for the shape and amplitude were minor. Testing lower cutoff frequencies of the bandpass filter up to 320 Hz primarily resulted in the removal of small peaks with high STDs, and the STDs were also reduced in general. With 320 Hz the detection rate remained similar to the results when using 40 Hz (after 10 strides 76.9, 88.1, and 96.5%, after 40 strides 90.4, 97.3, and 99.6% for 2, 4, and 8 kg, respectively) while the long-term drift was largely removed. After an initial reduction during the first 4 min of walking, for each of the sessions 2–4, MAV_int_ of the unloaded condition remained at about 92% (at 320 Hz). As drift occurs at low frequencies, this can be minimized using such a filtering approach. While the drift reduction did not further improve the detection rates, it would improve the ability for HITL comparisons of early and late assistance patterns over 1 h of optimization. As we found no other reference for the explored behavior, we would recommend assessing whether a similar EMG behavior exists for other data sets as well.

We decided to select and mainly publish results with the 40 Hz lower cutoff frequency for the bandpass filter, as this covers most of the identified improvements due to the artifact and drift removal.

## 5. Conclusion

This study explored the mean absolute values (MAV) of lower limb EMG during walking during loaded and unloaded conditions, with the aim of using the MAVs for HITL optimization. We found that transition strides between the loaded and unloaded conditions have increased MAV amplitudes compared to the previous and following steady-state conditions, which could cause a misinterpretation of the condition comparison in HITL optimization. As we aimed to compare steady gait conditions, we excluded 30 transition strides from each analyzed transition. However, based on literature and visual observation of the individual conditions, the exclusion of 3–20 strides seems sufficient for transition scenarios when changing assistance patterns in lower limb wearable robots. Further, we found it is worthwhile to combine the MAVs of multiple strides for both the reference condition and the comparison to improve the detection rates for changes in EMG. Based on our findings we recommend using the mean MAV of at least 10 strides. To increase sensitivity, 40 strides or more can be beneficial. Thus, based on the 20 strides for the transition and the 10 strides for the observation, we recommend approximately 16.5 s in total as the time window per observation for HITL optimization. This is less than half of that mentioned by Han et al. (2021) and would allow for nearly seven times the number of testable parameter sets compared to using the metabolic cost (Zhang et al., 2017; Ding et al., 2018). Next, if possible, it is worthwhile to include multiple lower limb muscles as they can additionally improve the detection rate. The use of multiple strides and multiple muscles benefits from the same mathematical behavior, that being an increase in the precision of the compared values in combination with a decrease in the SD for the reference and the comparison condition. We found that the reduction of the SD is a key element to improve the detection rates. Additionally, while the effects on the overall study were low, we would recommend excluding EMG signal artifacts including distinct long-term changes with deficient data, strides with unreasonable short peaks, and strides with unreasonably large MAVs.

This study also explored the drift of the MAVs over the course of the whole experiment. We found that a large drift exists within the Warmup and after the breaks. We would recommend performing at least 6 min of Warmup to reduce the effect of drift and avoid breaks within the HITL optimization. The lower cutoff frequency of the bandpass filter turned out to be a great modulator to eliminate artifacts as well as the MAV long-term drift. Without eliminating the drift, we would not recommend comparing the absolute values of the early MAVs to MAVs in later stages of a HITL experiment as the change in the MAV due to drift is larger than the change in the MAV when transitioning from unloaded walking to loaded walking with our largest load evaluated of 8 kg.

## Data availability statement

All the data relevant to this study are presented within the manuscript. The datasets used and/or analysed during the current study are available from the corresponding author on reasonable request.

## Ethics statement

The studies involving human participants were reviewed and approved by the Institutional Review Board, Technical University of Darmstadt. The patients/participants provided their written informed consent to participate in this study.

## Author contributions

MG was responsible for funding acquisition, analyzed the data and prepared the related figures, and wrote the original draft. MG, JZ, FW, and GZ were responsible for the experimental design. MG, JZ, and FW performed the experiments. All the authors interpreted the data. and provided critical feedback on the manuscript.

## Funding

MG was funded by the German Science Foundation (DFG) under the Grant No. GR 4689/3-1 and the Technische Universität Darmstadt by the Athene Young Investigator Funding (No. 52700967). The funding for JZ was provided by the German Science Foundation (DFG, www.dfg.de) under Grant No. KO 1876/15-1. We acknowledge support from the German Research Foundation and the Open Access Publishing Fund of the Technical University of Darmstadt for covering publication costs. None of the funding sources had any influence on the design of the study and collection, analysis, and interpretation of data.

## Conflict of interest

The authors declare that the research was conducted in the absence of any commercial or financial relationships that could be construed as a potential conflict of interest.

## Publisher's note

All claims expressed in this article are solely those of the authors and do not necessarily represent those of their affiliated organizations, or those of the publisher, the editors and the reviewers. Any product that may be evaluated in this article, or claim that may be made by its manufacturer, is not guaranteed or endorsed by the publisher.
